# Fecal lactoferrin in discriminating inflammatory bowel disease from Irritable bowel syndrome: a diagnostic meta-analysis

**DOI:** 10.1186/1471-230X-14-121

**Published:** 2014-07-07

**Authors:** Xing-lu Zhou, Wen Xu, Xiao-xiao Tang, Lai-sheng Luo, Jiang-feng Tu, Chen-jing Zhang, Xiang Xu, Qin-dong Wu, Wen-sheng Pan

**Affiliations:** 1 Department of Gastroenterology, Second Affiliated Hospital of Zhejiang University, School of Medicine, 88 Jiefang Road, Hangzhou, China; 2 Department of Pharmacy, Second Affiliated Hospital of Zhejiang University, School of Medicine, 88 Jiefang Road, Hangzhou, China

**Keywords:** Fecal lactoferrin, Inflammatory bowel disease, Irritable bowel syndrome, Meta-analysis

## Abstract

**Background:**

To perform a meta-analysis evaluating the diagnostic ability of fecal lactoferrin (FL) to distinguish inflammatory bowel disease (IBD) from irritable bowel syndrome (IBS).

**Methods:**

The Medline, EMBASE, Web of Science, Cochrane library and CNKI databases were systematically searched for studies that used FL concentrations to distinguish between IBD and IBS. The sensitivity, specificity, and other diagnostic indexes of FL were pooled using a random-effects model.

**Results:**

Seven studies, involving 1012 patients, were eligible for inclusion. In distinguishing IBD from IBS, FL had a pooled sensitivity of 0.78 (95% confidence interval [CI]: 0.75, 0.82), a specificity of 0.94 (95% CI: 0.91, 0.96), a positive likelihood ratio of 12.31 (95% CI: 5.93, 29.15), and a negative likelihood ratio of 0.23 (95% CI: 0.18, 0.29). The area under the summary receiver-operating characteristic curve was 0.94 (95% CI: 0.90, 0.98) and the diagnostic odds ratio was 52.65 (95% CI: 25.69, 107.91).

**Conclusions:**

FL, as a noninvasive and simple marker, is useful in differentiating between IBD and IBS.

## Background

Inflammatory bowel disease (IBD) and irritable bowel syndrome (IBS) are common conditions that may present with a similar symptom complex of abdominal pain and altered bowel habits. However, the two conditions differ markedly in their pathophysiology, prognosis and therapeutic approaches. IBD represents a group of idiopathic, chronic, inflammatory intestinal conditions [1], commonly requires a lifetime of medical care, and can even cause significant morbidity. In contrast, IBS is a chronic, functional gastrointestinal disorder without inflammation [2], with most IBS patients having a favorable prognosis. Despite existing diagnostic criteria, such as the Rome criteria, it remains difficult to distinguish IBD from IBS using symptoms and signs only, especially in the absence of rectal bleeding and systemic illness. As clinical differentiation remains challenging and may delay effective treatment, most patients with IBS are evaluated by endoscopy and radiographic imaging to exclude a diagnosis of IBD. This not only exposes patients to the inherent risks associated with these procedures, but increases their economic burden [3]. Less invasive and less expensive diagnostic methods are needed to more effectively rule out IBD in primary care patients with chronic abdominal complaints.

Fecal measurements of potential biomarkers are noninvasive diagnostic tests for intestinal mucosal inflammation and may correlate well with disease activity. Several neutrophil-granular proteins released by activated neutrophils may constitute fecal markers of intestinal inflammation, including lactoferrin (LF), calprotectin (Cal), polymorphonuclear neutrophil-elastase (PMN-e), and lysozyme (Lys) [4], with Cal and LF appearing to be the most promising surrogate biomarkers. Cal is a calcium- and zinc-binding protein that constitutes up to 60% of the total cytosolic protein content of neutrophils [5]. Cal has been shown to reflect neutrophil migration in the intestines of IBD patients and measurements of this protein may be an alternative to ^111^indium labeled radioactive techniques [6]. LF is an iron binding glycoprotein with a molecular mass of about 80 kDa that is present in various secretory fluids, such as milk, saliva, tears, and nasal secretions [7]. LF is a component of the innate immune system, with antimicrobial activity as a bactericide and fungicide, as well as being a major constituent of neutrophil granules that is released during apoptosis [8]. During intestinal inflammation polymorphonuclear neutrophils infiltrate the mucosa, increasing LF concentration in feces proportional to neutrophil translocation to the GI tract [9]. Studies investigating whether FL can be used as a noninvasive marker to distinguish IBD from non inflammatory conditions, especially IBS, have yielded variable results [3,10-18]. We therefore designed a meta-analysis to assess the overall diagnostic capacity of FL in discriminating IBD from IBS.

## Methods

### Literature search

Medline (using PubMed as the search engine), EMBASE, Web of science, Cochrane library and China National Knowledge Infrastructure (CNKI) databases were searched by two reviewers (XZ and WX) independently for relevant articles published in English and Chinese up to November 2013. The MeSH headings and key words used were "fecal lactoferrin", "lactoferrin and inflammatory bowel disease", "lactoferrin and irritable bowel syndrome", "IBD and IBS", and "lactoferrin and intestinal inflammation". Further searches included combinations of "lactoferrin" with "enteritis", "ileitis", "enteritidis", and "esoenteritis". We also screened the reference lists of included studies and review articles. The results were then hand searched for eligible studies.

### Study eligibility

Studies were included if they met the following criteria: 1) assessed the diagnostic performance of FL in discriminating IBD from IBS; 2) used endoscopic and histological methods as the reference standards; 3) presented sufficient information to calculate the true-positive (TP), false-positive (FP), true-negative (TN), and false-negative (FN) rates; and 4) were conducted on human subjects including pediatric or adult population. Letters, reviews, conference abstracts, comments and case reports were excluded because of the limited data presented. When the same population was reported in two or more publications, only the most informative or complete study was included to avoid duplication of information.

### Data extraction and quality assessment

The same reviewers (XZ and WX) independently retrieved specific data from each full-text article using a standard data extraction form, which included author, year of publication, nation, patient characteristics, FL assay, and cutoff value. The published values for TP, FP, TN, and FN were extracted and used to construct a 2 × 2 contingency table. Disagreement between the two reviewers was resolved by consensus. If consensus among the two reviewers could not be reached, a third investigator (XT) was referred to for arbitration and consensus. The methodological quality of the included studies was evaluated using the Quality Assessment of Diagnostic Accuracy Studies (QUADAS) list [19], which consists of 14 items scored as "yes", "no" or "unclear".

### Statistical analysis

Statistical analyses were performed using Meta-Disc software version 1.4 (for Windows; XI Cochrane Colloquium; Barcelona, Spain) and STATA statistical software version 12.0 (StataCorp; College Station, TX, USA). For each study, the following indexes of test accuracy were computed by constructing a 2 × 2 contingency table: sensitivity, specificity, positive likelihood ratio (PLR), negative likelihood ratio (NLR), and diagnostic odds ratio (DOR). A random-effects model was used to calculate the pooled diagnostic indices, with corresponding 95% confidence intervals (CIs), across studies. A summary receiver operating characteristic (sROC) curve was plotted to determine the relationship between sensitivity and specificity, and the area under the curve (AUC), an analytical summary of the test performance, was determined, with the upper and lower limits of the 95% CI of the AUC calculated as AUC + 1.96 Se and AUC–1.96 Se, respectively. Heterogeneity was assessed by determining the Cochran Q-statistic and by the test of inconsistency (*I*^*2*^) [20], with a *P*-value < 0.05 or *I*^*2*^ > 50% suggesting significant heterogeneity [21]. A random effects model, which considered both within- and between-study variations [22], was used throughout because of the observational nature of most studies. In addition, the Spearman’s rank correlation coefficient was calculated to assess the threshold effect. Finally publication bias was determined using Egger precision weighted linear regression tests [23].

## Results

**Figure 1:**
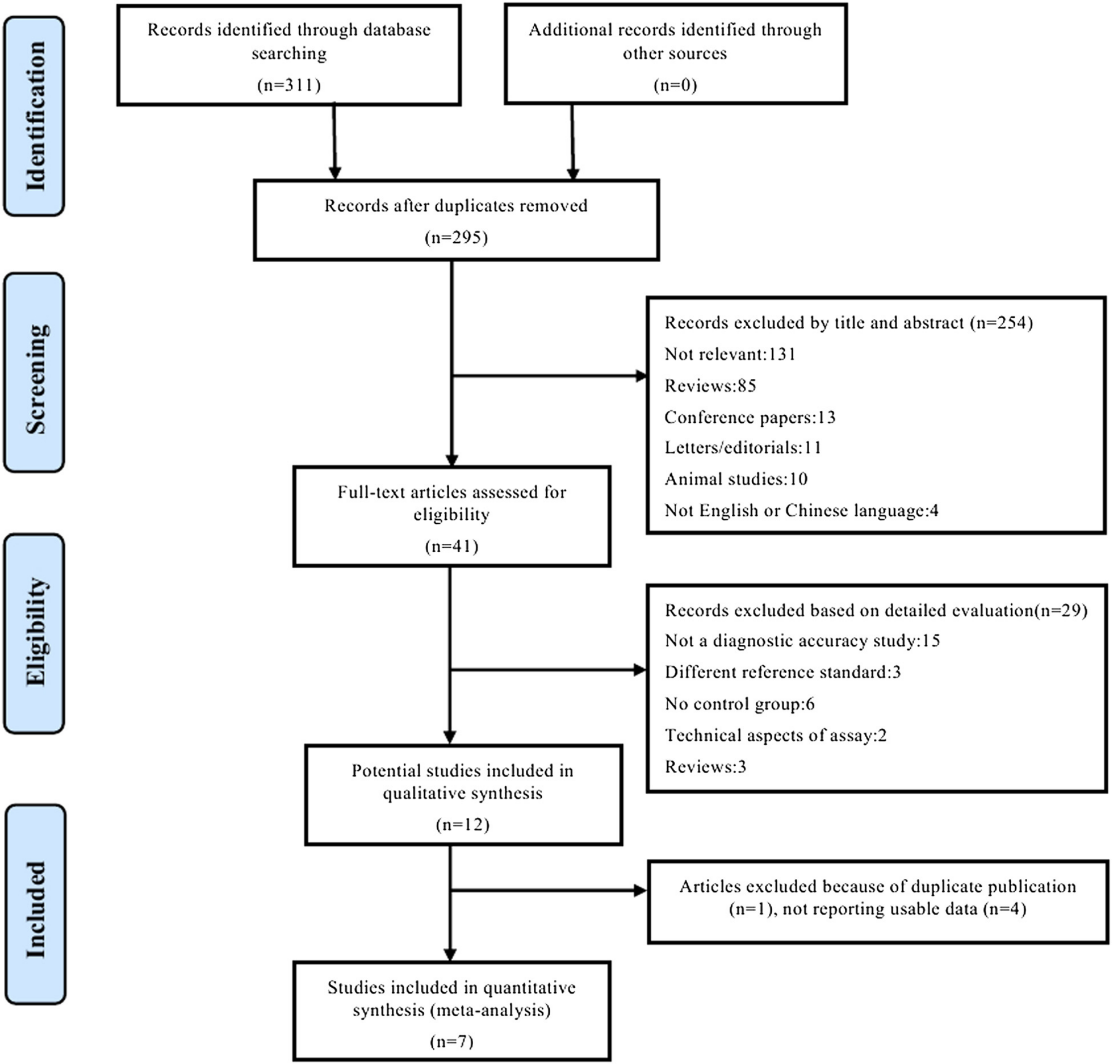
Flowchart of the study selection process.

The initial literature search identified a total of 311 reports; of these, 270 were excluded based on their titles and abstracts (Figure 1). A review of the full text of the remaining 41 articles led to an additional 34 being excluded because of failure to meet the inclusion criteria, duplicate publication or not reporting the data required to create a contingency table. Finally, seven eligible studies were included [3,10-15] (Table 1).

**Table 1:** Characteristics of the studies included in the meta-analysis

**Study**	**Year**	**Age(mean/range)**	**Nation**	**Number of patients**					**FL assay**	**Cut off**	**Test result**				**SEN (95% CI)**	**SPE (95% CI)**
				**IBD**	**UC**	**CD**	**IBS**	**control**			**TP**	**FP**	**FN**	**TN**		
Walker	2007	13.4/2-21	American	141	62	79	7	22	IBD-SCAN	7.25 ug/ml	118	1	23	28^‡^	0.84 (0.77-0.89)	0.97 (0.82-1.00)
Schoepfer	2008	20-79	Switzerland	64	28	36	30	42	IBD-SCAN	7.0 ug/ml	55	1	9	29	0.86 (0.75-0.93)	0.97 (0.83-1.00)
Langhorst	2008	15-70	Germany	85	42	43	54	-	IBD-SCAN	7.05 ug/ml	68	1	17	45	0.80 (0.70-0.88)	0.83 (0.71-0.92)
Kane	2003	10-78	American	149	71	78	31	56	IBD-SCAN	4 ug/g	111	0	38	31	0.74 (0.67-0.81)	1.00 (0.89-1.00)
Schroder	2007	20-75	Germany	45	20	25	31	-	IBD-SCAN	7.3 ug/g	37	0	8	31	0.82 (0.68-0.92)	1.00 (0.89-1.00)
Sidhu	2010	42/58/56*	UK	230	126	104	137	98	IBD-SCAN	7.25 ug/g	70^§^	6	32	131	0.69 (0.59-0.77)	0.96 (0.91-0.98)
Otten	2008	52.3/44.5^†^	Netherlands	23	-	-	91	-	IBD-SCAN	7.25 mg/ml	18	9	5	82	0.78 (0.56-0.93)	0.90 (0.82-0.95)

*Numbers are mean values for IBS, UC and CD respectively. ^†^Numbers are mean values for IBS and IBD respectively.

^‡^Include 22 healthy volunteers. ^§^Only calculated active IBD.

**Figure 2:**
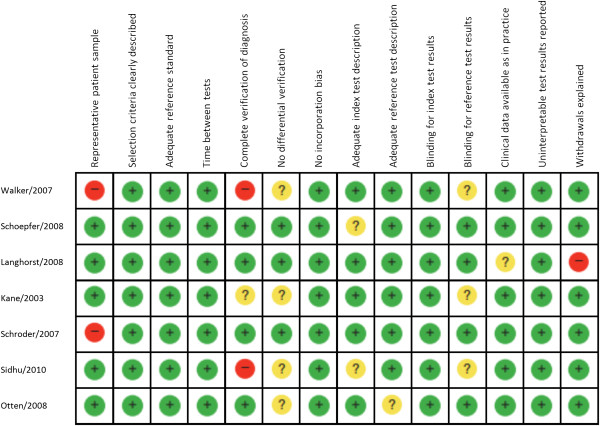
**Methodological quality of each included study.** +, Yes; -, No; ?, unclear.

**Figure 3:**
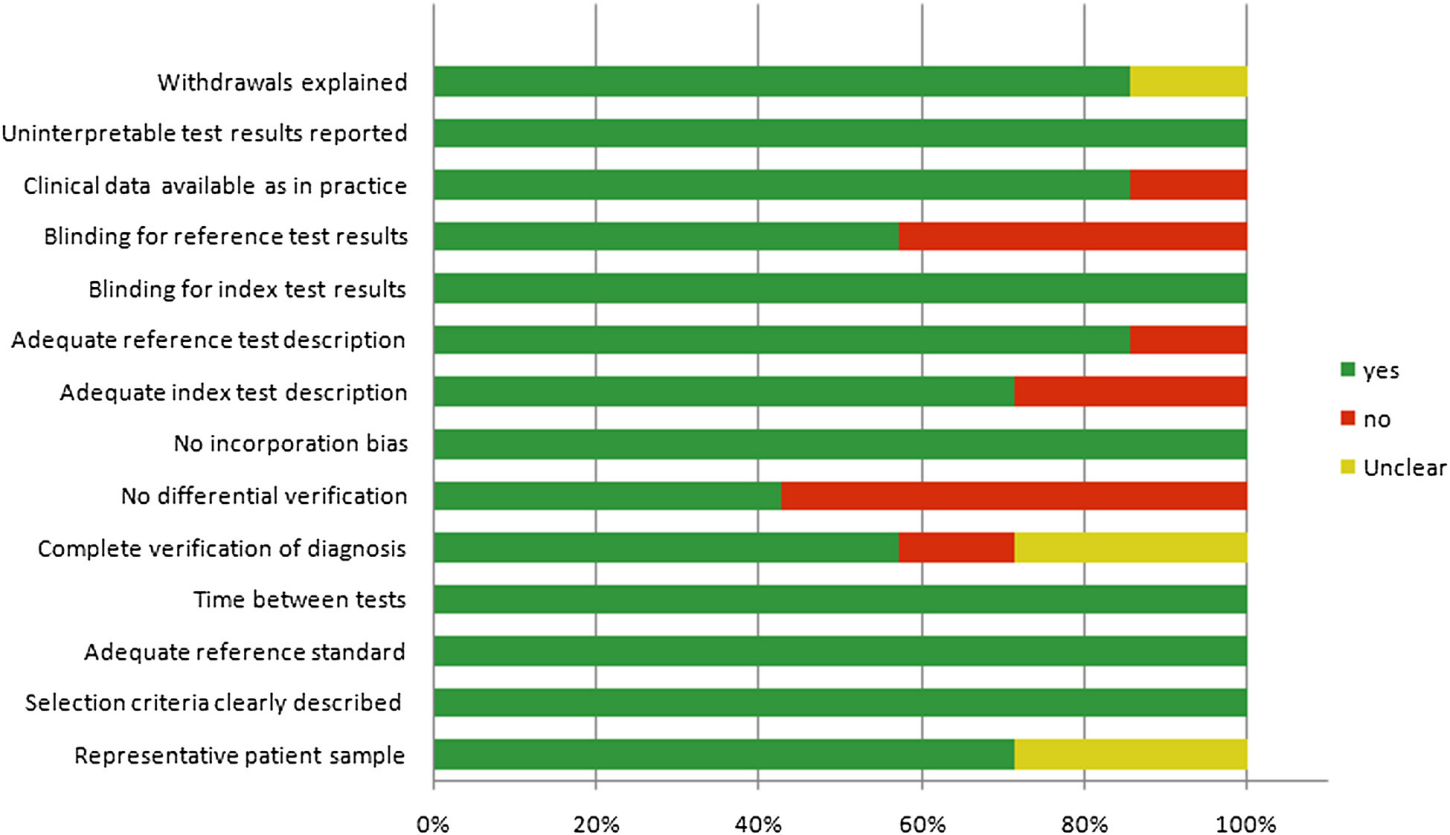
Assessment of methodological quality items shown as percentages across all included studies.

The seven eligible studies included a total of 1336 patients, including 737 with IBD, 381 with IBS, and 218 healthy volunteers, who underwent FL testing. One study found that mean FL level was similar in patients with IBS and healthy control; that study combined these two groups for comparisons with patients with IBD [10]. Another study only reported the TP, FP, TN, and FN rates of FL assay in distinguishing active IBD from IBS [14]. As our aim was to observe the diagnostic performance of FL in differentiating IBD from IBS, our meta-analysis included 1012 patients, 609 with IBD, 381 with IBS and 22 healthy volunteers. Of the seven included studies, one measured FL solely in children and young adults [10], three measured FL in both children and adults [12-14], and the other three measured FL in adults only [3,11,15]. All seven studies used a commercially available enzyme-linked immunosorbent assay (IBD-SCAN®, Techlab, Blacksburg, VA, USA) to measure FL. The methodological quality of the included studies is presented in Figure 2. Assessment of each risk of bias item is shown as a percentage across all of the included studies (Figure 3).

**Figure 4:**
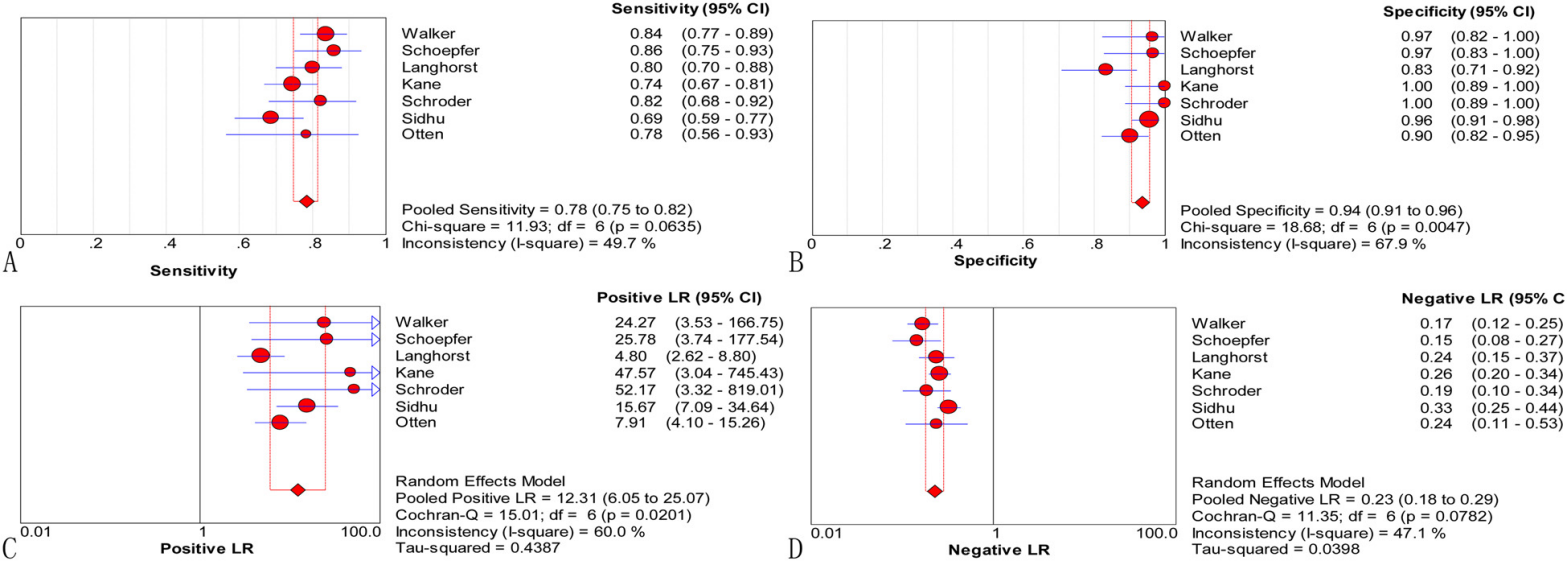
**Forest plots of sensitivity (A), specificity (B), positive likelihood ratio (C), and negative likelihood ratio (D) with corresponding 95% CIs of FL in distinguishing IBD from IBS.** The size of the solid circle indicates the effect size of each study.

**Figure 5:**
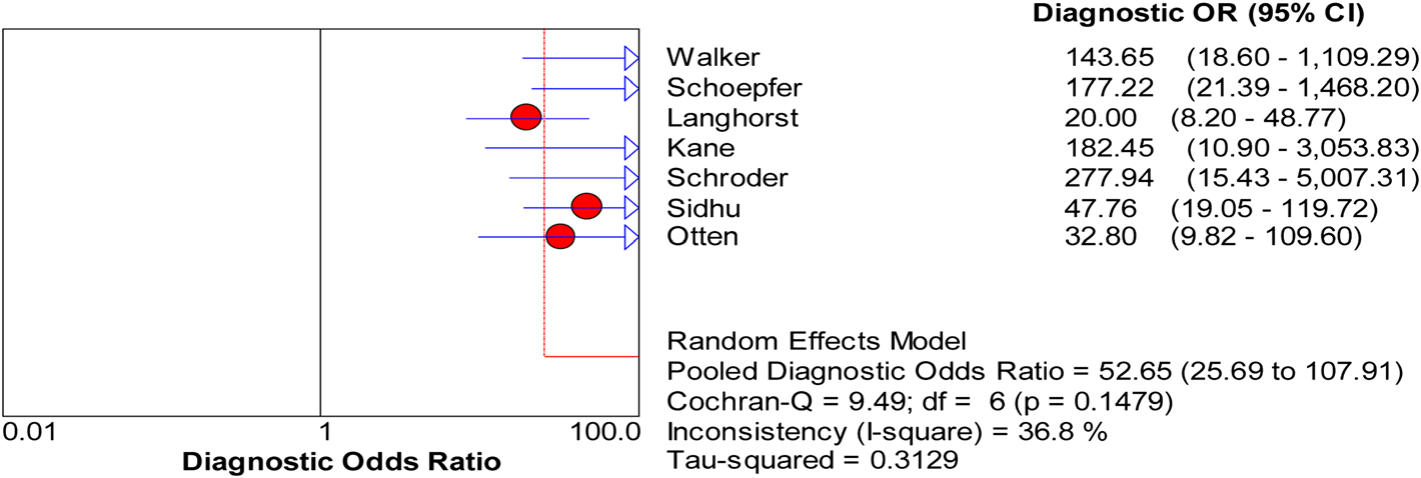
Forest plots of diagnostic odds ratio with corresponding 95% CIs of FL in distinguishing IBD from IBS.

Figure 4(A) shows the Forest plot for sensitivity and Figure 4(B) shows the Forest plot for specificity of FL in discriminating between IBD and IBS. Sensitivities ranged from 0.69 to 0.86 (pooled sensitivity 0.78; 95% CI: 0.75, 0.82) and specificities from 0.83 to 1.00 (pooled specificity 0.94; 95% CI: 0.91, 0.96). The pooled PLR was 12.31 (95% CI: 6.05, 25.07; Figure 4(C)), the pooled NLR was 0.23 (95% CI: 0.18, 0.29; Figure 4(D)), and the pooled DOR was 52.65 (95% CI: 25.69, 107.91; Figure 5). The chi-square values for sensitivity, specificity, PLR, NLR and DOR were 11.93 (*P* = 0.063), 18.68 (*P* = 0.005), 15.01 (*P* = 0.020), 11.35 (*P* = 0.078), and 9.49 (*P* = 0.148), respectively and their *I*^*2*^ values were 49.7%, 67.9%, 60.0%, 47.1%, and 36.8%, respectively. Significant heterogeneity among were observed for specificity and PLR.

**Figure 6:**
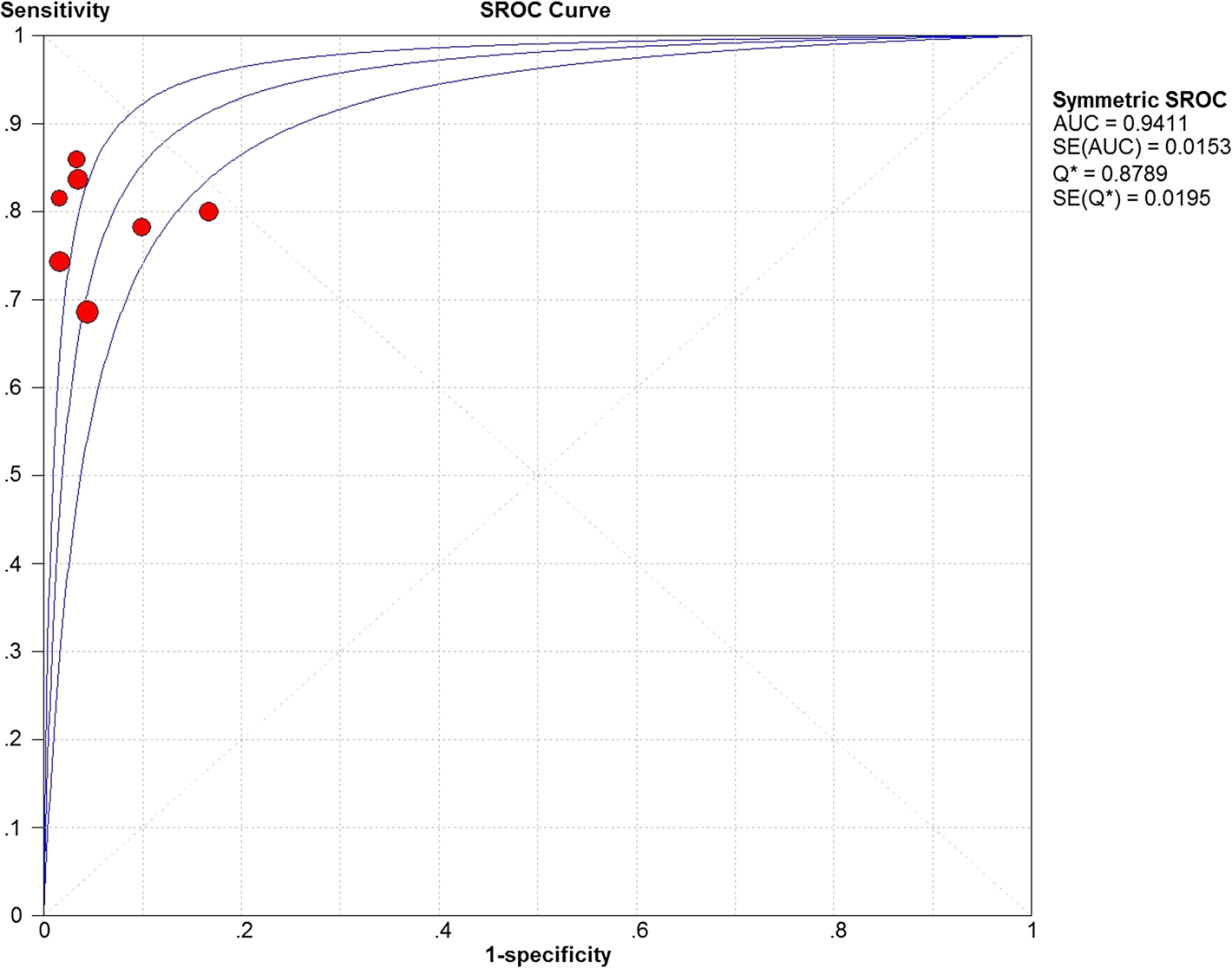
**Summary ROC curves for FL in discriminating IBD from IBS.** The size of the solid circle represents the sample size of each study included in the meta-analysis. The regression sROC curves summarize the overall diagnostic accuracy

The Spearman’s rank correlation coefficient, performed as a test for threshold effect, was found to be -0.198 (*P* = 0.670), indicating that no threshold effect could have caused variations in accuracy estimates among the individual studies.The pooled sROC (Figure 6) showed an AUC of 0.94 with a standard error (SE) of 0.02. The 95% CI was 0.90–0.98, while the pooled diagnostic accuracy (Q*) was 0.88 (SE 0.01), demonstrating that FL was highly able to differentiate between IBD and IBS.

The Egger test showed that the potential publication bias was significant (*P* < 0.05). Owing to the limited number of studies included in our meta-analysis, funnel plots were not assessed.

## Discussion

FL assays may detect mucosal inflammatory activity at a level insufficient to cause increases in erythrocyte sendimentation rate (ESR) and C-reactive protein (CRP). FL levels are apparently unaffected by a variety of non-intestinal conditions that elevate markers of systemic inflammation [24]. Lactoferrin is highly stable in feces for up to 7 days at room temperature [9], enabling patients to conveniently collect samples at home and transport them to distant hospitals or laboratories [5]. Moreover, quantitative FL levels can be easily and reliably measured by commercially available enzyme-linked immunoabsorbent assay kits that have been approved by the US Food and Drug Administration [5,25]. FL concentrations have been found to correlate well with endoscopic and histological activity [4,26-29]. Moreover, FL measurements can be used to evaluate responses to anti TNF-α treatment [30,31] or to predict relapse in IBD patients [10,32]. Studies have sought to evaluate the accuracy of FL in the diagnosis of organic (versus functional) intestinal diseases, mainly to discriminate IBD from non inflammatory conditions, especially IBS. Few studies to date have directly compared the diagnostic performance of fecal lactoferrin and Cal tests, with some reporting that both are similar in their ability to detect intestinal inflammation [11,12,29], while others found that Cal [3,17] or FL [33] is more accurate diagnostically. LF seems to be more stable at room temperature and therefore may be preferred if long delays are expected before analysis. Age-related variability seems to be less pronounced with lactoferrin and therefore may be preferable in pediatric patients [34]. To our knowledge, this study is the first meta-analysis to assess the diagnostic performance of FL in differentiating between IBD and IBS.

The present meta-analysis demonstrated that FL level had a pooled sensitivity of 0.78 and a pooled specificity of 0.94 in distinguishing IBD (active and inactive) from IBS. The pooled sROC showed an AUC of 0.94, indicating a promising discriminative ability. The DOR, defined as the ratio of the odds of positive test results between the diseased and non diseased groups, with higher values indicating greater accuracy, was 52.65 [35]. Likelihood ratio is a metric that incorporates both the sensitivity and specificity, is less affected by prevalence, and is widely considered more useful in clinical practice [36]. Although there is no absolute threshold, a good diagnostic test may have a PLR >5 and an NLR <0.2 [37]. We observed a PLR of 12.31, indicating that patients with IBD had an approximately 12.31-fold higher chance of testing positive than patients with IBS. We also observed an NLR of 0.23, indicating that a patient with IBD had a 23% chance of testing negative.

The 1012 patients analyzed in our study included both children and adults. Although FL has been reported to vary by age in healthy volunteers [38], the results from the seven included studies showed no significant difference between children and adults. These findings suggested that FL assays could be utilized in a wide spectrum of age groups. Most of the included studies [3,10-14] contained patients with IBD of different disease severity, with FL being a more sensitive assay in patients with active than inactive IBD. Because the data were insufficient and the various studies utilized different clinical activity indices, our analysis failed to stratify patients by disease activity. Nevertheless, several studies have found that FL levels are higher in patients with inactive IBD than in patients with IBS [10,12-14].

Ulcerative colitis (UC) and Crohn’s disease (CD) are two types of IBD with different inflammatory patterns. UC is primarily characterized by superficial inflammation of the colon with neutrophil infiltration, the obligatory involvement of the rectum in some adult patients or the left colon, together with superficial inflammation, leading to a short transit time of released FL. In contrast, patients with CD show small intestinal involvement, with a longer transit time and/or the accumulation of inflammatory cells in deeper mucosal layers possibly resulting in FL levels being higher in CD than in UC [4,39]. In contrast, one study reported higher FL concentrations in CD [13], and another showed that FL levels tended to be higher in patients with isolated colonic disease than in patients with involvement of the ileum alone or the ileocolon [3].Owing to data limitations, we did not perform subgroup analysis based on disease type and location.

Our study had several limitations. First, all studies included in our meta-analysis were from western countries, which may have biased our findings. Second, our meta-analysis was based on published studies; the exclusion of unpublished data (grey literature) is generally associated with an overestimation of the true effect, thus resulting in a publication bias. Third, the reliability of the pooled estimates was dependent on the methodological quality of the included studies. Although eligible studies met many of the QUADAS criteria, weaknesses remained. Fourth, some degree of heterogeneity was introduced by the variability in patient characteristics. These individuals had different types of IBD, different disease activity and different sites of inflammation. Owing to the small number of people included in this meta-analysis and the lack of available information, we did not perform subgroup analysis by pretest probability of CD or UC (based on clinical assessment), or by disease severity or distribution. Fifth, one study included in our meta-analysis pooled data from IBS patients and healthy controls. Since healthy individuals were not representative of the study population, it may have generated some heterogeneity. Sixth, although all included studies measured FL using the same assay, the thresholds varied, perhaps owing to differences in subject profile or inclusion or exclusion criteria. Therefore, we could not determine an optimum cutoff value for FL.

## Conclusions

This meta-analysis showed that FL appears to have good diagnostic precision in distinguishing IBD from IBS both in adults and children. Owing to study limitations, additional high-quality original studies (especially in patients stratified by disease type, severity and distribution) are required to confirm the predictive value of FL.

## Competing interests

The authors declare that they have no competing interests.

## Authors' contributions

WP, XX and QWcontributed to the conception, design and final approval of the submitted version. XZ, WX and XT developed the literature search, carried out the extraction of data, assisted in the critical appraisal of included studies and assisted in writing up. LL, JT and CZ carried out the statistical analysis of studies. All authors read and approved the final manuscript.

## Pre-publication history

The pre-publication history for this paper can be accessed here:

http://www.biomedcentral.com/1471-230X/14/121/prepub
